# The Prevalence of Hypertension in the Population without Awareness of the Disease: Data from a Rural Town of Shandong Province, China

**DOI:** 10.1155/2021/9672994

**Published:** 2021-11-03

**Authors:** Maoti Wei, Li Dong, Fenghua Wang, Kai Cui, Jiamin Yu, Delong Ma, Ning Yang, Yuming Li

**Affiliations:** ^1^Center of Clinical Epidemiology, TEDA International Cardiovascular Hospital, Tianjin 300457, China; ^2^Center Hospital of Beikuo Town, Rizhao 276809, Shandong, China; ^3^Hospital of Gaoxing Town, Rizhao 276811, Shandong, China; ^4^Hospital of Lanshan District, Rizhao 276810, Shandong, China; ^5^Department of Hypertension, TEDA International Cardiovascular Hospital, Tianjin 300457, China; ^6^TEDA International Cardiovascular Hospital, Tianjin 300457, China

## Abstract

**Objective:**

To understand the prevalence of hypertension in the population without awareness of hypertension in a rural area, a cross-sectional study was carried out.

**Methods:**

Blood pressures were measured in residents over 60 years during the National Basic Public Health Service project carrying on in a rural town of Shandong province. Combined with detail information of the hypertension disease history, the status of prevalence of hypertension was calculated. Basic information and clinical laboratory examinations were analyzed with Student' *t* or *t*' or chi-square test for univariate analysis. Multinomial logistic analysis was used in exploring multiple variables.

**Results:**

According to the individual history and blood pressure levels, the awareness rate of hypertension in the population over 60 years old was 50.3% (1285/2554, 95% CI: 48.3–52.3%). The prevalence of hypertension was 55.1% (1270/2304, 95% CI: 53.1–57.2%) in the population without awareness of hypertension, in which the proportion of stage 1 hypertension was 58.8% (747/1270), stage 2 hypertension was 28.9% (367/1270), and stage 3 hypertension was 12.3% (156/1270). The prevalence of hypertension in men was 54.4% (611/1124, 95% CI: 51.4–57.3%), which was almost the same as that in women (55.8%, 659/1180, 95% CI: 53.0–58.7%) (*X*^2^ = 0.515, *P*=0.473). The prevalence of hypertension increased with age (*X*^2^_trend_ = 11.848, *P*=0.001). Age, BMI, total cholesterol, triglyceride, and drinking rate were positively correlated with the prevalence of hypertension, that is, the higher the level of these factors, the higher the prevalence of hypertension; on the contrary, LDL and smoking rate might be negatively correlated with the prevalence of hypertension, which means, the higher the prevalence of hypertension, the lower the level of these two indicators. Binary and multinominal logistic results showed that age, BMI, and drinking had stronger effects on the higher blood pressure level.

**Conclusions:**

The awareness rate of hypertension among the elderly in a rural area needs to be further improved. In the prevention and control of hypertension, close attention should be paid to the group of elder, high BMI index, high levels of total cholesterol and triglyceride, and drinking habits.

## 1. Main Text

Hypertension is one of the most common chronic diseases in China, and it is the most important risk factor of cardiovascular and cerebrovascular disease death in urban and rural residents [1]. Although hypertension can be prevented and controlled, it is necessary to know or get a diagnosis before taking control measures. The results of large-scale research in 28 provinces of China showed that the awareness rate of hypertension was 33.8% [2] and that in East China was 46.5% [3]. In rural regions, the awareness rate of hypertension increased from about 15% in 1991 to less than 30% in 2015 [4]. In addition to not knowing about their own hypertension, the rural population also know less about hypertension knowledge. The study shows that the overall awareness rate of hypertension patients was 47.60% [5]. It can be seen that both the awareness rate of hypertension and the awareness rate of hypertension knowledge in rural population are in the low level.

With the progress of National Basic Public Health Service in China, some rural areas had carried out physical examination for rural residents and intervention of some chronic diseases. By making full use of these basic data, it is expected to know the condition of hypertension prevalence in some rural areas and analyze the factors that may be related to hypertension to provide guidance for the further development of better health services.

## 2. Methods

### 2.1. Subjects and Information Collection

From February 2020 to December 2020, relying on the National Basic Public Health Service project, a township hospital in Shandong province carried out the health examinations for residents over 60 years old. The checkup collected 132 items including basic information, physical examination, blood biochemistry, ECG data, personal habits, disease history, treatments, and psychological status of the participants, and the participants were informed that the data may be used for scientific research purposes, and a personal data use license was obtained according to the principle of informed consent. For the sake of personal confidentiality, before professional analysis, the data were first checked and then decrypted. The study was approved by the ethics committee of the author's hospital.

**Figure 1:**
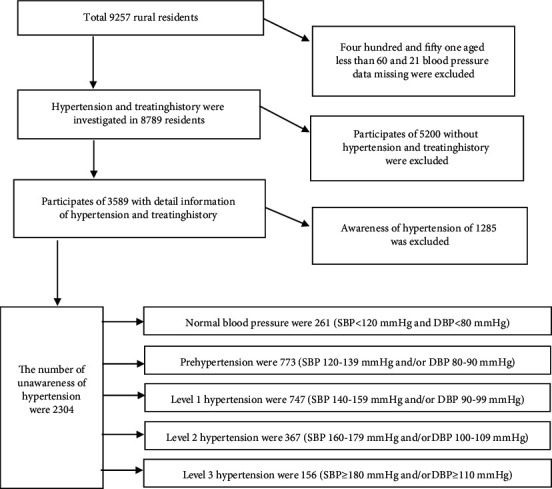
Data process flow for investigating the prevalence of hypertension without awareness of disease history in a rural area in Shandong province, China.

In physical examination, the blood pressure of the subjects was measured according to the blood pressure measurement standard, and the awareness of hypertension and medication were investigated. The analysis process of the subjects is shown in Figure 1.

### 2.2. Demographics

In this study, hypertension was diagnosed and determined according to the screening process and the key points and methods provided by the national guidelines for the prevention and treatment of hypertension at the population level (2020 Edition) [6]. In short, the diagnosis was determined according to the outpatient blood pressure combined with disease history. If the participants had the blood pressure agreeing with the standard of hypertension or were taking hypertension drugs, they were judged with hypertension. BMI was calculated by dividing the weight in kilograms by the height in meters squared.

### 2.3. Diagnostic Criteria

The diagnosis and staging of hypertension were based on the following criteria: prehypertension (systolic blood pressure 120–139 mmHg and/or 80–90 mmHg), stage 1 hypertension (systolic blood pressure 140–159 mmHg and/or 90–99 mmHg), stage 2 hypertension (systolic blood pressure 160–179 mmHg and/or 100–109 mmHg), and stage 3 hypertension (systolic blood pressure ≥180 mmHg and/or ≥110 mmHg). The diagnosis of hypertension includes 1–3 stages of hypertension. The standard of isolated systolic hypertension was systolic blood pressure ≥140–90 mmHg and diastolic blood pressure <90 mmHg.

Hypertension awareness is defined according to patients' self-report that they have been diagnosed with hypertension or are taking hypertension drugs. The awareness rate of hypertension was defined as the percentage of patients who knew they had hypertension before the investigation. The treatment rate was defined as the percentage of that among the hypertensive patients who had taken antihypertensive drugs or control methods in the last 2 weeks. Participants who had both SBP and DBP less than 140/90 mmHg were considered to have controlled hypertension, and the control rate was defined as the percentage of that among the hypertensive patients with controlled hypertension [7].

### 2.4. Data Processing and Statistical Methods

After establishing the database, data were cleaned for unqualified data before analysis. Quantitative data were described using mean ± standard deviation or median with quartile interval. Student's *t*-test or one-way ANOVA was used for the comparison between groups, and SNK (or LSD) was used for multiple comparisons among groups. Qualitative data (or counting data) were described as percentage or proportions, and comparison between groups was carried out using the chi-square test. Tests of two sides and *P* < 0.05 were statistically significant. Multinomial logistic regression analysis was used to analyze the factors related to hypertension levels. In the regression, the variable screening method was backward: conditional, the probability of a variable entering the equation was 0.05, excluding 0.10, and the other parameters were system default. The research data analysis and processing were completed by professional statistical analysts using SPSS software version 26.00 (Armonk, NY: IBM Corp).

## 3. Results

### 3.1. Prevalence of Hypertension in Rural Resident without Awareness of Hypertension

After measurement of blood pressure and acquirement of the medical history, the results could reflect the blood pressure control and awareness of the level of hypertension in the population.

Among the 3589 residents with information of the hypertension history and blood pressure control results, 2554 were found to have the hypertension history and/or blood pressure controlled. Among them, 1285 were clearly aware of hypertension, that is, the awareness rate of hypertension was 50.3% (1285/2554, 95% CI: 48.3–52.3%).

The prevalence of hypertension was 55.1% (1270/2304, 95% CI: 53.1–57.2%) in those without awareness of hypertension (Figure 1). The proportion of hypertension in stage 1 was 58.8% (747/1270), in stage 2 was 28.9% (367/1270), and in stage 3 was 12.3% (156/1270).

**Figure 2:**
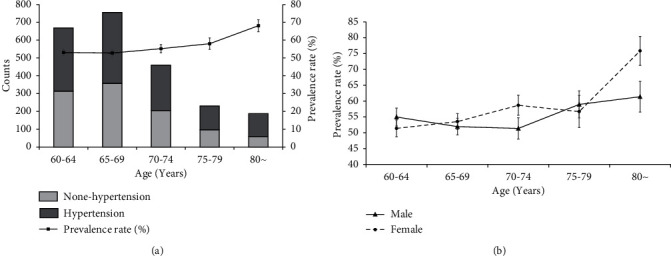
Prevalence of hypertension in a rural population without awareness of hypertension. (a) Prevalence of hypertension in different age groups. (b) Prevalence of hypertension in different gender and age groups.

The prevalence of hypertension increased with age (*X*^2^_age trend_ = 11.848, *P*=0.001) (Figure 2(a), Table 1). However, the prevalence was 54.4% (611/1124, 95% CI: 51.4–57.3%) in males and 55.8% (659/1180, 95% CI: 53.0–58.7%) in females, and there was no significant difference between the two groups (*X*^2^ = 0.515, *P*=0.473). However, the prevalence rate was 61.4% (62/101, 95% CI: 51.2–70.9%) in males and 75.9% (66/87, 95% CI: 65.5–84.4%) in females, with a significant difference between the two groups (*X*^2^ = 4.507, *P*=0.034) (Figure 2(b), Table 1).

### 3.2. Factors Related to Hypertension in Rural Residents without Awareness of Hypertension

The results showed that age, waist circumference, BMI, total cholesterol, and triglyceride were higher in the hypertension population than those of normal people (*t*′_age_ = 3.848, *P* < 0.001 ; *t*′_waist circumference_ = 5.959, *P* < 0.001; *t*_BMI_ = 8.736, *P* < 0.001; *t*_total cholesterol_ = 3.182, *P* = 0.001; *t*′_triglyceride_ = 4.291, *P* < 0.001). Waist circumference, BMI, total cholesterol, and triglycerides also increased with the increase of blood pressure level (Table 1).

Among the factors of individual behavior, the elder frequented 30% with smoking habits, of which the hypertension population rated 28.1%, lower than 32.3% of the none hypertension population (*X*^2^ = 4.692, *P*=0.030), and the smoking rate decreased with blood pressure level (*X*^2^ = 6.365, *P*=0.012). However, the drinking rate was 28.6%, among which the drinking rate of the hypertension group (31.9%) was higher than that of none hypertension group (24.6%) (*X*^2^ = 14.975, *P*=0.030), and the drinking rate increased with the blood pressure level (*X*^2^ = 4.889, *P*=0.027 ) (Table 1).

Binary logistic results showed that age, BMI, total cholesterol, triglycerides, and alcohol consumption were positively correlated with the prevalence of hypertension, that is, the higher the level or the proportion of these factors, the higher the prevalence of hypertension. On the contrary, LDL and smoking may be negatively correlated with hypertension, i.e., the higher the prevalence of hypertension were, the lower the level or proportion of these two indicators were (Table 2). When normal as a reference, multinomial logistic results showed that age, BMI, and drinking had stronger effects on the higher blood pressure level (Table 3).

## 4. Discussion

Primary medical and health institutions (community health service centers, community health service stations, township hospitals, and village clinics) are the “main battlefield” for hypertension management, and their management level will directly affect the development trend of cardiovascular and cerebrovascular diseases in China in the future. Report on cardiovascular health and disease in China (2019) shows that the number of hypertension in China has reached 245 million [8]. However, the first step of controlling hypertension is to diagnose it. Based on the National Basic Public Health Service project, this study used the health screening data in township hospitals to understand the prevention and control of hypertension in rural residents. The results showed that the awareness rate of hypertension in rural areas was 50.3% (1285/2554, 95% CI: 48.3%–52.3%), which was slightly higher than large-scale studies [3]. The town is located in a relatively developed economy region with relative sufficient medical resources. The process of urbanization in this region has been significantly accelerated, and the population has gradually changed from rural to urban residents. In this case, the disease occurrence mode has also changed rapidly, and the disease prevention awareness of the population is relatively strong, which may be the reason for the higher awareness rate of hypertension than other regions. However, the absolute number of the awareness rate is just over half, which reminds that nearly half of the people with hypertension have not been diagnosed and treated. There may be several reasons for the low awareness rate of hypertension. First, people do not pay attention to hypertension. At the rate of information answered by the residents, more than one-half of them knew little about their health problems or paid no attention to blood pressures before symptoms appeared, thus reducing the awareness rate of hypertension (Figure 1). Second, it may be due to the limited knowledge of hypertension and the ignorance of hypertension-related knowledge. For example, the survey results show that the awareness rate of hypertension knowledge in this province is 47.60% [5], which is lower than that in other provinces in China. Third, there may be a certain amount of masked hypertension. Data from Americans show that the prevalence of masked hypertension in adults is 12.3% and that in people over 65 years old is 28% [9].

Usually, in the prevention and control or intervention of hypertension, it is mainly aimed at people with confirmed hypertension. If the population were not screened and monitored, a large case number of hypertension would be missed, which greatly reduces the intervention effect for hypertension control. In this study, we found that the prevalence of hypertension was 55.1% (1270/2304, 95% CI: 53.1–57.2%) in the rural population without awareness of hypertension, i.e., if these cases were not treated, hypertension would bring great harms. Studies have shown that for every 10 mmHg decrease in systolic blood pressure or 5 mmHg decrease in diastolic blood pressure, the risk of death is reduced by 10–15%, the risk of stroke is reduced by 35%, the risk of coronary heart disease is reduced by 20%, and the risk of heart failure is reduced by 40% [6]. Therefore, the fact that the low awareness rate and the high prevalence rate of hypertension in the population without knowing the disease reminds primary medical institutions that they need to take more initiative to carry out hypertension monitoring and screening in this region to ensure the early detection of patients with hypertension and improve the awareness rate and management rate.

A large number of studies have shown that the prevalence of hypertension increases with age [4, 10–12]. The results of this study also showed that the prevalence of hypertension in different age groups over 60 years old increased with age (*X*^2^_trend_ = 11.848, *P*=0.001). At the same time, there was no difference in the prevalence of hypertension between men and women (*X*^2^ = 0.515, *P*=0.473 ). Waist circumference, BMI, total cholesterol, triglycerides, smoking, and drinking were closely related to hypertension. Further analysis showed that age, BMI, total cholesterol, triglycerides, drinking, and other factors were positively correlated with the prevalence of hypertension, that is, the higher the level or proportion of these factors, the higher the prevalence of hypertension. Low-density lipoprotein and smoking may be negatively correlated with hypertension, which means the higher the prevalence of hypertension, the lower the level or proportion of these two indicators. Multinomial results showed that age, BMI, and drinking had stronger effects on the higher level of blood pressure. Most of the results in this study were consistent with others [4], such as age, waist circumference, BMI, total cholesterol, triglycerides, and drinking. However, there were also some inconsistencies, for example, the relationship between smoking and hypertension. In this study, smoking was negatively correlated with hypertension. This trend has been also observed in other cohort observations [13]. Researchers have found that smoking is significantly correlated with masked hypertension [14], which often underestimates the role of smoking in hypertension.

## 5. Limitations

This study is a cross-sectional study, data on chronic diseases were self-reported, the prevalence of hypertension was calculated for the population, and the results may be underestimated. However, similar as many large-scale research results in China based on similar studies [3, 4, 10, 12], the results of this study need further confirmation. Therefore, a cohort could be built for exploring the incidence of diseases including hypertension and other noncommunicable chronic diseases.

In conclusion, according to investigating on the prevalence of hypertension among rural residents without awareness of hypertension in some areas of China, it is found that the awareness rate of hypertension is low and the prevalence rate of hypertension is high, which reminds us that the awareness rate of hypertension needs to be further improved in promoting the prevention and control of hypertension at the grassroots level (especially in areas from rural transform to urban). At the same time, close attention should be paid to those of elders, high BMI, high levels of total cholesterol and triglyceride, and with drinking habits.

## Figures and Tables

**Table 1:** The related factors of hypertension in the population without awareness of hypertension in a rural area over 60 years old.

	Normal	Prehypertension	Level 1	Level 2	Level 3	Statistics	*P*	Trend	*P*_trend_
Gender (male/female)	140/121	373/400	373/374	177/190	61/95	*χ* ^2^ = 8.842	0.065	*χ* ^2^ = 5.088	0.024
Age (years)	67.9 ± 5.6	68.5 ± 6.4	68.6 ± 6.3	69.9 ± 7.4	72.2 ± 8.7	*F* = 13.964	<0.001	*F* = 50.501	<0.001
Waist circumference (cm)	82.8 ± 11.0	85.8 ± 11.1	87.6 ± 11.8	88.7 ± 12.6	87.7 ± 12.0	*F* = 12.520	<0.001	*F* = 29.735	<0.001
BMI (kg/m^2^)	22.5 ± 3.0	23.5 ± 3.3	24.3 ± 3.5	24.7 ± 3.7	24.8 ± 3.8	*F* = 24.403	<0.001	*F* = 71.754	<0.001
TC (mmol/L)	5.4 ± 1.0	5.5 ± 1.0	5.6 ± 1.1	5.6 ± 0.9	5.6 ± 1.1	*F* = 2.884	0.021	*F* = 8.648	<0.001
TG (mmol/L)	1.1 ± 1.1	1.2 ± 0.9	1.3 ± 1.0	1.3 ± 0.9	1.6 ± 2.3	*F* = 7.202	<0.001	*F* = 24.462	<0.001
LDL-C (mmol/L)	2.4 ± 0.7	2.5 ± 0.7	2.5 ± 0.7	2.5 ± 0.7	2.5 ± 0.7	*F* = 1.268	0.280	*F* = 1.083	0.298
HDL-C (mmol/L)	1.9 ± 0.5	1.9 ± 0.5	1.9 ± 0.6	1.8 ± 0.6	1.9 ± 0.5	*F* = 0.539	0.707	*F* = 0.534	0.505
Smoke (yes/no)	88/168	242/525	213/528	107/260	35/121	*χ* ^2^ = 8.148	0.086	*χ* ^2^ = 6.365	0.012
Drink (yes/no)	50/211	204/569	245/512	121/246	39/117	*χ* ^2^ = 24.112	<0.001	*χ* ^2^ = 4.889	0.027
Healthy diet (yes/no)	252/9	740/33	713/34	353/14	153/3	*χ* ^2^ = 2.698	0.610	*χ* ^2^ = 0.819	0.365
Physical exercise (yes/no)	5/256	21/752	28/719	12/355	3/153	*χ* ^2^ = 3.324	0.489	*χ* ^2^ = 0.058	0.397
Comorbidity of cardiovascular and cerebrovascular diseases (yes/no)	16/245	46/727	41/706	17/350	6/150	*χ* ^2^ = 1.860	0.761	*χ* ^2^ = 1.839	0.175
Comorbidity of diabetes (yes/no)	32/221	82/656	93/640	38/311	20/127	*χ* ^2^ = 1.748	0.782	*χ* ^2^ = 0.043	0.813

Blood pressure classification: normal blood pressure with SBP<120 mmHg and DBP<80 mmHg, prehypertension with SBP 120–139 mmHg, and/or DBP 80–90 mmHg. Level 1 hypertension with SBP 140–159 mmHg and/or DBP 90–99 mmHg. Level 2 hypertension with SBP 160–179 mmHg and/or DBP 100–109 mmHg. Level 3 hypertension with SBP ≥ 180 mmHg and/or DBP ≥ 110 mmHg. BMI, body mass index; TC, total cholesterol; TG, triglyceride. LDL-C, low-density lipoprotein; HDL-C, high-density lipoprotein. Method = BSTEP (COND) age gender waist circumference TC TG LDL-C HDL-C smoke drink. Healthy diet physical exercise comorbidity of cardiovascular and cerebrovascular diseases comorbidity of diabetes.

**Table 2:** Binary logistic results of variables with hypertension in the population without awareness hypertension.

	Univariate		Multivariate^*∗*^	
	*P*	OR (95% CI)	*P*	OR (95% CI)
Gender (female, male as reference)	0.473	1.062 (0.901–1.252)		
Age (years)	<0.001	1.024 (1.012–1.037)	<0.001	1.038 (1.024–1.052)
Waist circumference (cm)	<0.001	1.022 (1.014–1.029)		
BMI (kg/m^2^)	<0.001	1.115 (1.087–1.144)	<0.001	1.129 (1.098–1.161)
TC (mmol/L)	0.002	1.141 (1.051–1.238)	0.001	1.287 (1.114–1.488)
TG (mmol/L)	<0.001	1.243 (1.117–1.383)	0.028	1.122 (1.012–1.244)
LDL-C (mmol/L)	0.203	1.080 (0.960–1.215)	0.007	0.749 (0.607–0.925)
HDL-C (mmol/L)	0.795	0.980 (0.842–1.141)		
Smoke (yes/no)	0.030	0.820 (0.685–0.981)	0.009	0.748 (0.602–0.929)
Drink (yes/no)	<0.001	1.438 (1.196–1.729)	<0.001	1.800 (1.443–2.245)
Healthy diet (yes/no)	0.955	1.012 (0.667–1.535)		
Physical exercise (yes/no)	0.224	1.359 (0.829–2.227)		
Comorbidity of cardiovascular and cerebrovascular diseases (yes/no)	0.316	0.832 (0.581–1.192)		
Comorbidity of diabetes (yes/no)	0.572	1.078 (0.832–1.396)		
Constant			<0.001	0.002

^*∗*^Note: multivariate program and parameters. Logistic regression variables hypertension./Contrast (male) = indicator (female)./Contrast (exercise) = indicator (no exercise)./Contrast (no smoke) = indicator (smoke)./Contrast (healthy diet) = indicator (no healthy diet)/Contrast (no drink) = indicator (drink)./Print=CI (95)./Criteria = pin (0.05) pout (0.10) iterate (20) cut (0.5).

**Table 3:** Multinomial logistic results of variables with hypertension in the population without awareness hypertension.

	Prehypertension		Level 1 hypertension		Level 2 hypertension		Level 3 hypertension	
	*P*	OR (95% CI)	*P*	OR (95% CI)	*P*	OR (95% CI)	*P*	OR (95% CI)
Intercept	0.048		<0.001		<0.001		<0.001	
Age (years)	0.054	1.024 (1.000–1.049)	0.003	1.039 (1.013–1.065)	<0.001	1.072 (1.043–1.101)	<0.001	1.120 (1.086–1.155)
Waist circumference (cm)	0.036	1.023 (1.001–1.045)	0.068	1.020 (0.999–1.043)	0.092	1.021 (0.997–1.046)	0.582	0.992 (0.963–1.021)
BMI (kg/m^2^)	0.103	1.056 (0.989–1.127)	<0.001	1.160 (1.087–1.238)	<0.001	1.208 (1.125–1.297)	<0.001	1.278 (1.178–1.388)
TC (mmol/L)	0.137	0.784 (0.568–1.081)	0.831	0.965 (0.697–1.336)	0.966	1.008 (0.699–1.454)	0.060	1.537 (0.983–2.403)
TG (mmol/L)	0.780	1.033 (0.825–1.293)	0.184	1.158 (0.933–1.437)	0.213	1.155 (0.920–1.449)	0.040	1.274 (1.011–1.605)
LDL-C (mmol/L)	0.057	1.536 (0.987–2.389)	0.499	1.167 (0.746–1.824)	0.691	1.107 (0.670–1.828)	0.066	0.562 (0.305–1.038)
HDL-C (mmol/L)	0.376	1.186 (0.812–1.733)	0.135	1.339 (0.913–1.963)	0.228	1.308 (0.846–2.023)	0.726	1.102 (0.641–1.894)
Gender, male	0.068	1.415 (0.974–2.056)	0.407	1.174 (0.803–1.715)	0.237	1.297 (0.843–1.995)	0.096	1.583 (0.921–2.722)
Diabetes, yes	0.431	0.836 (0.536–1.305)	0.969	0.991 (0.635–1.547)	0.469	0.826 (0.492–1.386)	0.982	1.007 (0.539–1.882)
Comorbidity of cardiovascular and cerebrovascular diseases, yes	0.634	0.864 (0.473–1.578)	0.474	0.797 (0.429–1.482)	0.202	0.621 (0.299–1.290)	0.148	0.480 (0.178–1.296)
Physical exercise, yes	0.627	1.284 (0.468–3.520)	0.380	1.563 (0.576–4.243)	0.568	1.381 (0.456–4.180)	0.877	0.889 (0.201–3.939)
Healthy diet, yes	0.643	0.826 (0.369–1.852)	0.468	0.740 (0.328–1.670)	0.855	0.916 (0.358–2.347)	0.690	1.324 (0.334–5.249)
Smoke, yes	0.626	0.909 (0.621–1.333)	0.063	0.690 (0.467–1.020)	0.176	0.735 (0.471–1.148)	0.071	0.575 (0.316–1.047)
Drink, yes	0.002	1.941 (1.278–2.946)	<0.001	2.857 (1.878–4.348)	<0.001	3.018 (1.890–4.822)	0.004	2.433 (1.329–4.453)

Note: multinomial program and parameters, the normal as reference. NOMREG hypertension level (base = first order = ascending) by gender diabetes comorbidity of cardiovascular and cerebrovascular diseases physical exercise healthy diet drink with age waist circumference BMITC TG LDL-C HDL-C./Criteria CIN (95) delta (0) MXITER (100) MXSTEP (5) CHKSEP (20) Lconverge (0) Pconverge (0.000001) singular (0.00000001)./Model./Stepwise = pin (.05) pout (0.1) mineffect (0) rule (none) entrymethod (LR) removalmethod (LR)./Intercept = include./print = parameter summary LRT step./Scale = 1.

## Data Availability

The data used to support the results of this study are available from the first author upon request.
